# Has vaccination alleviated the strain on hospitals due to COVID-19? A combined difference-in-difference and simulation approach

**DOI:** 10.1186/s12913-022-08541-x

**Published:** 2022-09-21

**Authors:** Mari Grøsland, Vilde Bergstad Larsen, Kjetil Telle, Hege Marie Gjefsen

**Affiliations:** Norwegian Institute of Public Health, Cluster for Health Services Research, Postboks 222, Skøyen, N-0213, 0473 Oslo, Norway

**Keywords:** COVID-19, Vaccine, Hospital capacity, Simulation model, Vaccine strategies

## Abstract

**Background:**

Serious measures, including mass vaccination, have been taken to ensure sufficient hospital capacity during the COVID-19 pandemic. Due to high hospitalization risk in the oldest age groups, most countries prioritized elderly for vaccines. The aim of this study is to broaden the understanding of how vaccination in younger age groups relieved the strain on hospitals during the pandemic.

**Methods:**

To determine the impact of vaccination on hospitalization, we relied on individual level data on health care use and vaccination from the Norwegian Emergency Preparedness Register Beredt C19. Using a pre-post design, we estimated the increase in hospitalization days from before to after confirmed COVID-19 for individuals aged 18-64 who were fully vaccinated (*N*=2 419) or unvaccinated (*N*=55 168) with comparison groups of vaccinated (*N*=4 818) and unvaccinated (*N*= 97 126) individuals without COVID-19. To evaluate whether vaccination itself contributed to a strain in hospitals, we use a similar design to study hospitalization rates before and after vaccination by comparing individuals vaccinated with the first dose (*N*=67 687) to unvaccinated individuals (*N*=130 769). These estimates were incorporated into a simulation of hospitalization days with different vaccine scenarios to show how the estimated results might have mattered for the hospitals and their capacity.

**Results:**

Hospitalization days increased by 0.96 percentage point each day during the first week and 1.57 percentage points during the second week after testing positive for COVID-19 for unvaccinated individuals. The corresponding increase was 0.46 and 0.32 for vaccinated individuals, i.e., a substantial difference. The increase was significantly higher for those aged 45-64 than for those aged 18-25. We find no increase in hospitalization days due to vaccination. Simulation results show that vaccination reduced hospitalization days by 25 percent, mainly driven by age 45-64.

**Conclusion:**

Our findings indicate that vaccination of individuals aged 18-64 did alleviate pressure on hospitals. Whereas there was a substantial relieve from vaccinating the 45-64 age group, there was no such contribution from vaccinating the 18-25 age group. Our study highlights how simulation models can be useful when evaluating alternative vaccine strategies.

**Supplementary Information:**

The online version contains supplementary material available at 10.1186/s12913-022-08541-x.

## Background

Maintaining sufficient hospital capacity has been a key concern during the Coronavirus Disease 2019 (COVID-19) pandemic. To ensure hospitals’ ability to provide sufficient levels of treatment and handle a potential influx of COVID-19 patients, governments worldwide have implemented strict infection control measures and initiated mass vaccination to limit the spread of SARS-CoV-2.

Although overall infection rates have generally been higher for young adults in Norway, the single most important predictor of COVID-19 resulting in hospitalization or death is high age [1–3]. Vaccines were therefore prioritized to individuals of older age and medical risk groups, as well as to health care workers, to limit and relieve the capacity strain on hospitals [4]. In Norway, local areas associated with higher risk of infection spread was also prioritized [5]. Vaccination accelerated during the spring of 2021 in Europe and North America and has proven very effective in avoiding serious COVID-19 among vaccinated individuals, despite the emergence of more contagious virus variants, such as the Alpha and Delta variants [6–8]. Observational studies on vaccine effectiveness have suggested that mRNA vaccines reduce hospitalization with more than 85 percent for the Alpha and Delta variant [9, 10], while vaccine efficacy has been shown to be even higher [11]. However, vaccine effectiveness varies between population groups [12]. In the Norwegian context, Veneti et al. (2022) show vaccine effectiveness against infection with the Delta variant of 64 % for fully vaccinated individuals, and no significant difference in vaccine effectiveness between the Alpha and Delta variant [13]. Moreover, vaccinated individuals who got infected with COVID-19 had also lower probability of being hospitalized than unvaccinated individuals [8]. Counterfactual modelling studies have indeed shown that the number of hospitalizations due to COVID-19 was far lower in the spring of 2021 than it would have been without vaccination [14, 15]. However, a better understanding of the impact of vaccination on hospitalization rates, and thus the pressure on the health care systems is essential for policy makers. This study provides knowledge about how alternative vaccine strategies might alleviate the pressure on hospitals. It highlights trade-offs available to policy makers when deciding how to distribute vaccines.

By October 2021, the entire adult population had been offered at least one dose of a COVID-19 vaccine in Norway, and the vaccine uptake among these was around 90% [16]. Despite a very high vaccine uptake, the uncertainty about the threshold of herd immunity makes it important to evaluate different alternatives regarding further vaccine strategies and progress. Additionally, new virus variants develop for which existing vaccines might be less effective, as seen for the Omicron variant. Given limited supply of effective vaccines, countries worldwide risk facing difficult prioritization of vaccines all over again. In this study, we contribute to the assessment of how vaccination relieved the burden on hospitals by estimating the difference in hospitalization days before and after confirmed COVID-19 for both unvaccinated and vaccinated individuals. To explore whether vaccination itself could matter for hospital capacity we additionally estimated the difference in hospitalization days before and after vaccination. To assess whether the direct effect of vaccination on hospitalization differs with age, we run separate regressions for different age groups.

Furthermore, we incorporated these estimates into a micro simulation model that estimated number of hospitalization days from January 1st 2021 to October 30^th^, 2021 using four different scenarios: 1) without any vaccination in the period, 2) with everyone being vaccinated the whole period, 3) a gradual increase in the vaccination rates equal the observed vaccination status in Norway in the period of study, and 4) an alternative vaccine strategy that solely prioritizes by decreasing age. Our simulation approach highlights the magnitude of the estimates for the given population, and describe to what extent different vaccination strategies matter for the strains on hospitals using estimated causal changes at the individual level.

## Methods

### Data

We utilized nation-wide individual-level data from the BEREDT C19 register^1^, a newly developed emergency preparedness register aiming to provide rapid knowledge about the COVID-19 pandemic in Norway. From within BEREDT C19, we utilized data originating from the following registries: The National Population Register; Norwegian Patient Register to identify date and duration of hospitalizations and the degree of urgency for the admission; the Norwegian Immunization Register to find date of vaccination; and the Norwegian Surveillance System for Communicable Diseases to determine date of confirmed COVID-19 cases. Individuals could be linked across data sources and over time using an encrypted version of a unique personal identification number provided every resident of Norway.

### Study sample

Our study population of interest included all Norwegian residents aged 18-64 years with a permanent identification number, known county of residence and who had not tested positive for COVID-19 prior to January 1^st^, 2021 (Table 1). The adult population we study is interesting, as vaccine uptake and coverage might be more subject to different prioritization strategies and vaccine campaigns. The population 65+ is not included in this analysis. These are individuals with increased risk of hospitalization and severe illness due to COVID-19 and has therefore been prioritized for vaccines in most countries. Support and uptake in this specific group of the population has also been very high [16].

**Table 1:** Inclusion and exclusion criteria

	A) Confirmed COVID-19 (unvaccinated)	B) Confirmed COVID-19 (vaccinated)	C) Vaccinated
Study population	Age: 18-64 Norwegian residents No COVID-19 prior to January 1^st^, 2021	Age: 18-64 Vaccinated with at least two doses Norwegian residents No COVID-19 prior to January 1^st^, 2021	Age: 18-64 Norwegian residents No COVID-19 prior to January 1st, 2021
Inclusion criteria for treatment group	Confirmed COVID-19 cases between January 1st 2021 and August 1st 2021	Confirmed COVID-19 cases at least 7 days after second vaccination dose between January 1st and September 1st 2021	Ten percent^a^ of the individuals among the 20-40 percentile that got the vaccine first in the age categories:18-24, 25-39, 40-44, 45-64 ^b^
Eligible for comparison group	The full study population, (except those in the treatment group)	The full study population (except those in the treatment group)	The full study population (except the vaccinated in the treatment group and the vaccinated among the 20 percentile)
Excluded individuals and observations	Individuals who died, emigrated or were vaccinated before (hypothetical) treatment date were excluded entirely (C=10 421, T= 57). Individuals that died, emigrated, vaccinated or tested positive for COVID-19 after the treatment date were observed only until the date of this event (Person days: 123 678).	Individuals who died, emigrated or tested positive for COVID-19 before (hypothetical) treatment date (C=19, T=3). Individuals that died, emigrated, vaccinated or tested positive for COVID-19 after the treatment date were observed only until the date of this event (Person days: 154).	Individuals who died, emigrated or tested positive for COVID-19 before (hypothetical) treatment date (C=6 143, T= 770). Individuals that died, emigrated, vaccinated or tested positive for COVID-19 after the treatment date were observed only until the date of this event (Person days: 976 482).

^a^Only look at 10 percent of this sample to minimize strain on the computing capacity, as the matching procedure is computer-intensive

^b^The treatment group only includes individuals in the 20-40 percentile that was vaccinated first in each age group as the 20 percent that was vaccinated first was more likely to be prioritized for vaccination due to risk factors. Additionally, we exclude the 40-100 percentile from the treatment group as these was included in the target set used to construct a comparison group

We defined three mutually exclusive treatment groups consisting of individuals who were A) unvaccinated and who had confirmed COVID-19, B) vaccinated and who had confirmed COVID-19 after the second dose and C) vaccinated with the first dose of a COVID-19 vaccine (Table 1). Individuals who had both confirmed COVID-19 (sample A) and were vaccinated (sample C) during the study period were defined by the first occurrence and observed until the second occurrence. We then constructed three comparison groups using propensity score matching: one for unvaccinated with confirmed COVID-19 (A), one for those with confirmed COVID-19 after two doses of vaccine (B) and one for those that is vaccinated (C) (see Supplemental material for more details on the propensity score matching procedure). Everyone in the comparison groups were assigned a hypothetical treatment date equal to the date of confirmed COVID-19 or vaccination, respectively, of the individual they were matched to.

#### Outcomes

We studied all-cause hospitalization days (prevalence), restricted to acute inpatient care. We organized our data in a relative-day structure with date of confirmed COVID-19/date of vaccination set as the point of reference (*day zero*), respectively, and followed each patient for 28 days prior to and 28 days after day zero. The outcome variable was coded one from the date of admission up to and including the discharge date and zero otherwise. Vaccines should not be administered to individuals with infection and fever on or 10 days before the vaccination day, and we therefore excluded observations from 10 days before to 5 days after date of vaccination, as this otherwise may lead to selection of somewhat healthier individuals into our treatment groups.

#### Statistical analyses

To contribute to the assessment of how hospital capacity due to hospitalization of COVID-19 was relieved by vaccination, we calculated and plotted the daily hospitalization rates, which equals the number of individuals that were inpatient at a hospital (prevalence) on the given day, divided by the number of person days, from 28 days prior to and after day zero (date of confirmed COVID-19/vaccination). We did this separately for the three treatment and three comparison groups, as defined above.

Furthermore, we studied how hospital strain was affected after COVID-19/vaccination in separate analyses using an event study design operationalized as a generalized difference-in-difference (DiD) approach. DiD analyses evaluate the effect of an event (i.e., COVID-19 or vaccination) by comparing the change in the outcome for the affected group before and after the event, to the change over the same time span in a group not affected by the event [17–20]. It can be summarized with the following equation:

$$DiD=\left({\overline{Y}}_T^{Post}-{\overline{Y}}_T^{Pre}\ \right)-\left({\overline{Y}}_C^{Post}-{\overline{Y}}_C^{Pre}\ \right)$$

Where $\overline{Y}$ is the average rate of hospitalization in a given period after (post) or prior to (pre) confirmed COVID-19/vaccination for the treatment (T) and comparison (C) groups. In this study, the pre period included the 28 days prior to confirmed COVID-19/vaccination, while the post period was four separate 7-day periods (hereafter called week 1, week 2, week 3 and week 4) after COVID-19/vaccination.

We thus generalized the traditional DiD method by extending from one period before and one after the event (confirmed COVID-19/ vaccination) to four separate estimates for each week after the event, comparing to the hospitalization days in the 28-day period before [17–22]. In addition to this model with separate estimates for each week after day zero, we also estimated more detailed models, presented graphically, with separate estimates for each relative day prior to and after day zero and compared this to the hospitalization days on the day prior to date of confirmed COVID-19/vaccination. All DiD results was estimated using linear regression models with clustering on the individual level and presented as a difference in percentage points.

To investigate group differences, we also split the sample into four mutually exclusive age groups based on age prioritizations in the Norwegian vaccination program; 18-24, 25-39, 39-44, and 45-64, and did the regressions with DiD-specifications as described above.

To quantify to what degree the burden on hospitals was relieved by vaccination, we incorporated our DiD-estimates into a model that simulated what the hospitalization days would be in four different scenarios; 1) without any vaccination in the period, 2) with everyone being vaccinated the whole period, 3) with the actual vaccine roll-out in the period of study, and 4) an alternative roll-out that solely prioritizes by decreasing age, where we redistribute vaccines so that those in the age group 45-65 got their first and second dose before those in the age group 40-44 and so on (Supplemental figure S1). We did this in total and per age group.

The simulation is based on the same population as the DiD-analysis. We use aggregate data from data from October 1^st^, 2020 to October 30^th^, 2021 and estimate infection rates for the age groups in the analysis, controlling for age group, vaccination share within the age group, infection rates in the preceding week and calendar fixed effects. We then simulate infection rates from January 1^st^ to October 30^th^ based on these estimates under the assumption that no one are vaccinated. We then use the estimates from the DiD analyses to calculate hospitalization rates under the four scenarios:

$$\mathrm{Scenario}\ 1:\kern0.75em {\mathrm{I}}_{\mathrm{t},\mathrm{a}\kern0.75em }\mathbf{x}\kern0.5em {\beta}_{\mathrm{C}19-\mathrm{unvaccinated},\mathrm{a}}$$

$$\mathrm{Scenario}\ 2:\kern0.75em {\mathrm{I}}_{\mathrm{t},\mathrm{a}\kern0.5em }\mathbf{x}\ \left(1-\mathrm{VE}\right)\mathbf{x}\ {\beta}_{\mathrm{C}19-\mathrm{vaccinated},\mathrm{a}}$$

$$\mathrm{Scenario}\ 3\ \mathrm{a}\mathrm{nd}\ 4:{\mathrm{I}}_{\mathrm{t},\mathrm{a}\kern0.75em }\mathbf{x}\kern0.5em {\beta}_{\mathrm{C}19-\mathrm{unvaccinated},\mathrm{a}}\mathbf{x}{\mathrm{V}}_{\mathrm{t},\mathrm{a}}+{\mathrm{I}}_{\mathrm{t},\ \mathrm{a}}\mathbf{x}\ \left(1-\mathrm{VE}\right)\mathbf{x}\ {\beta}_{\mathrm{C}19-\mathrm{vaccinated},\mathrm{a}}\mathbf{x}\left(1-{\mathrm{V}}_{\mathrm{t},\mathrm{a}}\right)$$

Where I_t,a_ is the infection rate in week t age group a, β_C19-unvaccinated,a_ and β_C19-vaccinated,a_ are the effect estimates after COVID-19 infection for unvaccinated and vaccinated individuals respectively, for each age group a. VE is the vaccine effectiveness against the Delta variant of SARS-CoV-2 infections on 64.4 [13] , while V _t,a_ is the cumulative share if individuals vaccinated with two doses in week t in age group a in the actual and alternative vaccine roll-out. We did this for each of the four weeks after COVID-19 infection/vaccination, using the DiD-estimates for each week after infection/vaccination. 

All analysis were run in STATA 16.

## Results

### Impact of COVID-19 on all-cause acute overnight hospitalization among unvaccinated individuals in total and per age group

**Fig. 1:**
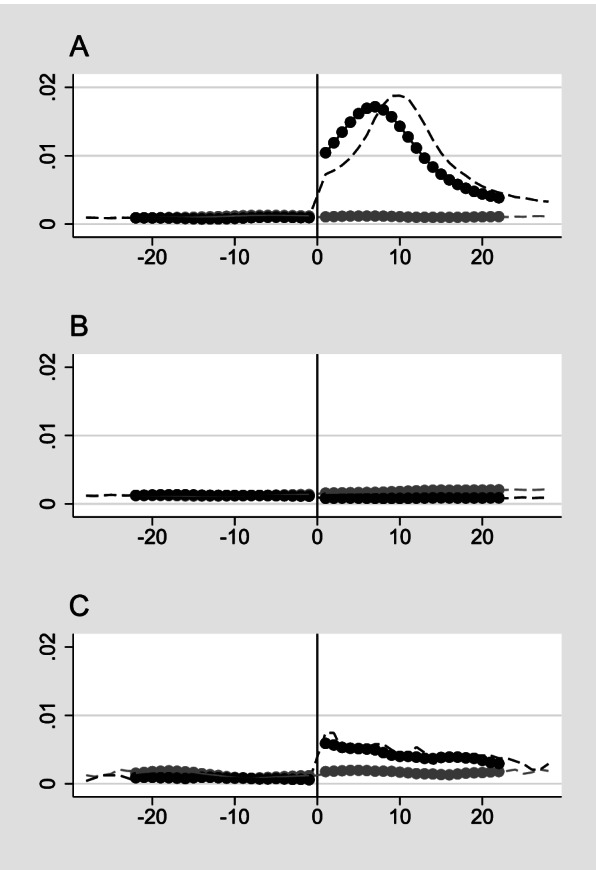
Impact of COVID-19 and vaccination on hospitalizations. Daily (dashed lines) and 7-day rolling average (solid lines) of daily acute overnight hospitalizations before and after the (hypothetical) treatment date of A) testing positive for COVID-19 (unvaccinated) B) testing positive for COVID-19 (vaccinated with two doses), and C) vaccinated with the first dose. The 7-day rolling average was calculated separately before and after the treatment date to visualize the change in health care use after vaccination. For days prior to the treatment date, the 7-day rolling average equals the mean of acute hospitalization the given day and the six preceding days, while for the days after treatment date equals the mean of health care use the given day and the six subsequent

Among 3 363 036 persons aged 18-64, we studied the treatment group of 55 168 unvaccinated individuals with confirmed COVID-19 and compared them with 97 126 matched individuals (sample A). As intended, there were only small differences on variables predicting positive COVID-19 test between the treatment and comparison group (Table 2, column A). In the 28-day (pre) period before confirmed COVID-19 the daily hospitalization rates for unvaccinated individuals was 0.11 for the comparison group and 0.09 in the treatment group. In week 1 and 2 after (hypothetical) date of confirmed COVID-19, the daily hospitalization rates remained unchanged for the comparison group, while it for increased from 0.09 to 1.04 and 1.67 for the treatment group (Fig. 1A, Table 2, column A).

**Table 2:** Descriptive statistics

	A) Confirmed COVID-19 for the unvaccinated		B) Confirmed COVID-19 for the vaccinated		C) Vaccinated (first dose)	
	Comparison Mean(sd)	Treatment Mean(sd)	Comparison Mean(sd)	Treatment Mean(sd)	Comparison Mean(sd)	Treatment Mean(sd)
Age						
18-24	0.26	0.26	0.17	0.17	0.11	0.11
	(0.44)	(0.44)	(0.38)	(0.38)	(0.31)	(0.32)
25-39	0.36	0.36	0.24	0.24	0.33	0.34
	(0.48)	(0.48)	(0.43)	(0.43)	(0.47)	(0.47)
40-44	0.10	0.10	0.11	0.11	0.10	0.10
	(0.30)	(0.30)	(0.38)	(0.32)	(0.30)	(0.30)
45-64	0.28	0.28	0.47	0.47	0.45	0.45
	(0.45)	(0.45)	(0.50)	(0.50)	(0.50)	(0.50)
Male	0.55	0.540	0.40	0.40	0.52	0.52
	(0.50)	(0.50)	(0.49)	(0.49)	(0.50)	(0.50)
Foreign born	0.41	0.41	0.36	0.36	0.18	0.19
	(0.49)	(0.49)	(0.48)	(0.48)	(0.39)	(0.39)
N:	97 126	55 168	4 818	2 419	130 769	67 687
Person days:	5 580 034	3 142 516	274 892	137 862	6 477 633	3 857 877
Daily hospitalization rates						
Pre	0,11	0,09	0,13	0,08	0,13	0,12
Week 1	0,11	1,04	0,18	0,59	0,16	0,08
Week 2	0,12	1,67	0,18	0,45	0,17	0,08
Week 3	0,10	0,73	0,13	0,38	0,20	0,09
Week 4	0,11	0,39	0,18	0,30	0,21	0,09

*SD* standard deviation

Our DiD estimates show that this corresponds to a 0.96 ((1.04 - 0.09) - (0.11 - 0.11)) percentage points increase the first week after testing positive for COVID-19 among the unvaccinated, compared to the matched control group (Table 3, column A). The increase was higher in the second week (1.57 percentage points) while the increase was smaller in the third and fourth week after date of confirmed COVID-19 (0.65 and 0.3 percentage points). Estimates on the daily difference in hospitalization days between those testing positive and the comparison group rose from the first date after testing positive, peaking after around 10 days (Supplemental Figure S2A).

**Table 3:** Impact of confirmed COVID-19 and COVID-19 vaccination on hospitalization

	A) COVID-19 (unvaccinated) b/(se)	B) COVID-19 (vaccinated) b/(se)	C) Vaccinated (first dose) b/(se)
Week 1	0.96^***^	0.46^***^	-0.07^***^
	(0.034)	(0.129)	(0.014)
Week 2	1.57^***^	0.32^**^	-0.09^***^
	(0.048)	(0.121)	(0.016)
Week 3	0.65^***^	0.30^*^	-0.11^***^
	(0.033)	(0.128)	(0.017)
Week 4	0.30^***^	0.17	-0.12^***^
	(0.026)	(0.119)	(0.018)
N:	8 722 550	412 754	10 335 510

Difference-in difference estimated for the change in acute overnight hospitalizations before and after treatment date. Standard errors (se) are clustered on individuals. The pre-period is reference period in all regressions. Significance levels: *<0.1, **<0.05, ***<0.01

**Fig. 2:**
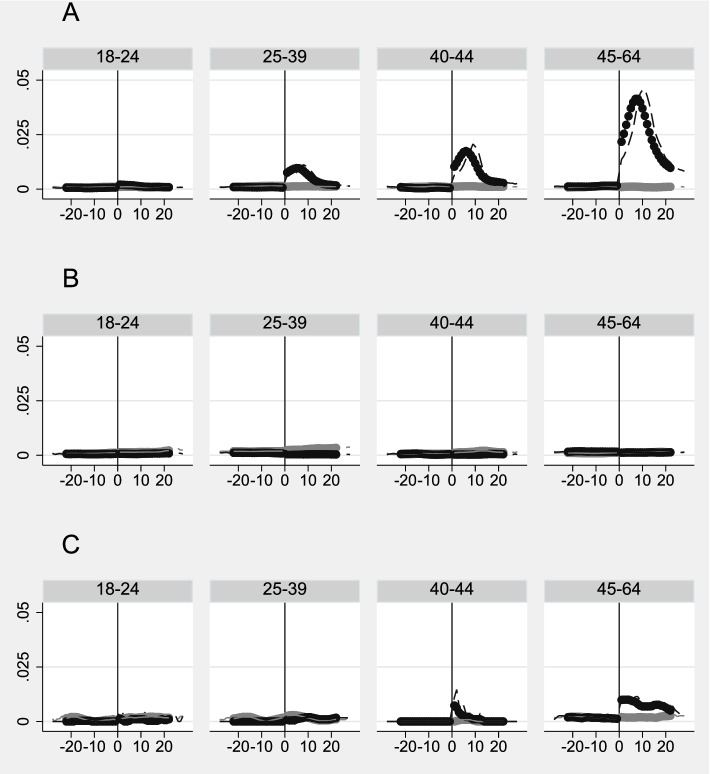
Impact of COVID-19 and vaccination on hospitalizations, by age groups. Daily (dashed lines) and 7-day rolling average (solid lines) of daily acute overnight hospitalizations before and after the (hypothetical) treatment date of A) testing positive for COVID-19 (unvaccinated), B) testing positive for COVID-19 (vaccinated with two doses) and C) vaccinated first dose for treatment group (black) and comparison group (grey). The 7-day rolling average was calculated separately before and after the treatment date to visualize the change in health care use after vaccination. For days prior to the treatment date, the 7-day rolling average equals the mean of acute hospitalization the given day and the six preceding days, while for the days after treatment date equals the mean of health care use the given day and the six subsequent

Hospitalization days after COVID-19 for unvaccinated individuals was found to differ between different age groups (Fig. 2A, Supplemental Figure S3 A). While those aged 18-24 only had a 0.14 percentage points increase in hospitalization days in week 1 the corresponding increase was 2.04 percentage points for those aged 45-64 (Supplemental Table S1, column A). The difference between the youngest and oldest age groups was greatest in the second week after positive test, where the number of hospitalization days was 50 (4/0.08) times larger for those aged 45-64 compared to those aged 18-24 (Fig. 2A, Supplemental Table S1, column A).

### Impact of COVID-19 on all-cause acute overnight hospitalization among vaccinated in total and per age group

For the sample of individuals testing positive for COVID-19 after two doses of vaccination and its matched comparison group (sample B) hospitalization remained stable in the comparison group (*N*=4 818) from the pre period to week 1-4 after date of confirmed COVID-19, while it increased from 0.08 to 0.59 from the pre period to week 1 after date of confirmed COVID-19 in the treatment group (*N*=2 419) (Fig. 1B, Table 2, column B). Estimation results shows that individuals with confirmed COVID-19 after two doses of COVID-19 vaccination had a 0.46 percentage points increase in hospitalization days one week after date of confirmed COVID-19 compared to their matched comparison group. The increase was lower in week two (0.32 percentage points) and three (0.3 percentage points) before it was no longer statistically significant in week four (Table 3, column B). Estimates on the daily difference in hospitalization days between those testing positive for COVID-19 after two doses of vaccination and the comparison group was highest right after date of confirmed COVID-19 and decreased evenly before it was back on pre level after around 27 days (Supplemental Figure S2B). Hospitalization after COVID-19 for vaccinated individuals also differed by age (Fig. 2B, Supplemental Figure S3B). Those aged 45-64 had the highest increase in hospitalization days. The difference between the youngest and oldest age groups was greatest in the first week after positive test, where the hospitalization days was 8 (0.8/0.1) times larger for those aged 45-64 compared to those aged 18-24 (Supplemental Table S1, column B)

### Impact of vaccination (first dose) on all-cause overnight hospitalization in total and per age group

Among the vaccinated individuals (*N*= 67 687) there was a slight decrease in hospitalization days from the pre period to the weeks after vaccination (Table 2, column C), which may indicate some selection of healthier individuals into vaccination. Our estimation results show that vaccinated individuals did not have increased numbers of hospitalization days in the weeks following vaccination relative to the comparison group (Fig. 1C, Table 3, column C), and this was also the case when we stratified our sample by age (Fig. 2C, Supplemental Table S1, column C)

### Simulation results

**Fig. 3:**
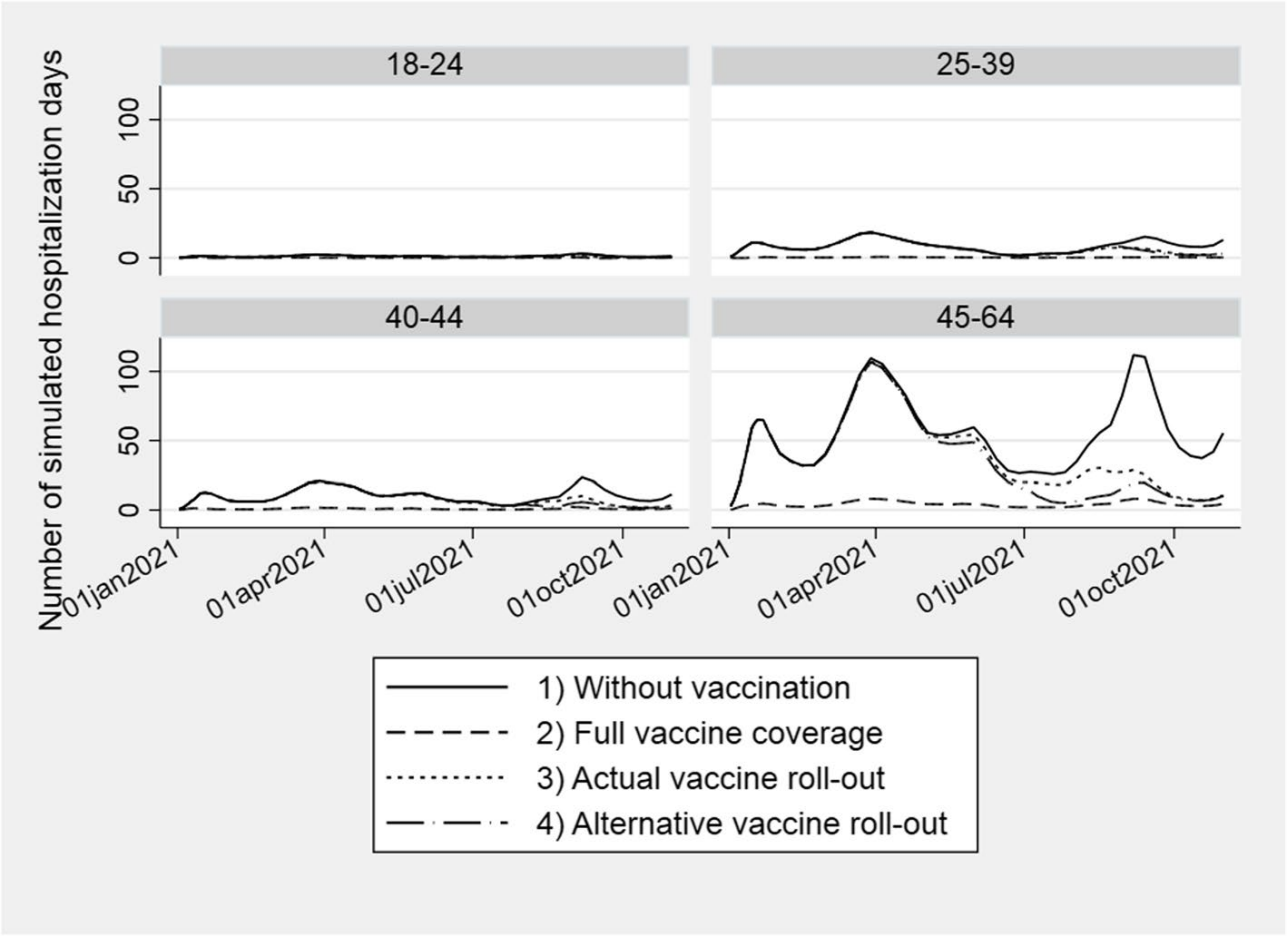
Simulation results for hospitalization days. Simulated hospitalization days based on estimates on the effect on hospitalization days after COVID-19 for unvaccinated vs. vaccinated individuals (Table 3, column A and B), the actual and alternative vaccine roll-out (Supplemental Figure S1), and the simulated infection rates for each age category (Supplemental Figure S4)

The point estimates above show substantially higher hospitalizations rates after detection of COVID-19 infection for unvaccinated individuals than for vaccinated individuals, and that vaccination itself does not increase the probability of being hospitalized (Table 3). Thus, the results indicate that vaccination against COVID-19 indeed reduce the burden on hospitals. To better understand the magnitude of these effects, we ran a simulation model where we predicted infection rates (Supplemental Figure S4) in a scenario without any vaccination, excluding contagion as a channel for effects of vaccination. We then used the estimates in Table 3 column A and B to predict hospitalization days in the three scenarios 1) without any vaccination in the period 2) with everyone being vaccinated the whole period 3) actual vaccine uptake in the period and 4) an alternative vaccine roll-out that solely prioritizes by decreasing age (Supplemental Figure S1). Without vaccination (scenario 1), we estimated that a total of 25 067 hospitalization days would have occurred between January 1^st^ and October 31^st^, 2021.The scenario with full vaccine coverage (scenario 2) is unrealistic in the period of study due to limited availability on vaccines. However, it illustrates the magnitude to which vaccination may reduce the burden on hospitals in the future, if such limitations no longer exist. Clearly, our results shows that full vaccine coverage reduce the burden on hospital (Fig. 3, Table 4). For the actual vaccine roll-out (scenario 3) we estimated that a total of 18 713 hospitalization days would occur. Hence, the vaccination campaign averted 6 354 hospitalization days corresponding to an average reduction in hospitalization days during the evaluation period on 25 percent compared to a situation without vaccination (Table 4). This number is conservative as we only consider the effect of two vaccine doses. In a situation with an alternative vaccine roll-out (scenario 4), the estimated hospitalization days was 17 242, corresponding to a reduction of 8 percent compared to the actual vaccine roll-out (scenario 3).

**Table 4:** Impact of COVID-19 vaccination on all-cause hospitalization days

	Number of hospitalization days				% Change	
	Scenario 1) Without vaccination	Scenario 2) Full vaccine coverage	Scenario 3) Actual vaccine roll-out	Scenario 4) Alternative vaccine roll-out	Without vaccination vs. actual vaccine roll-out	Actual vaccine roll-out vs. alternative vaccine roll-out
Age 18-24	404	16	326	389	19	-19
Age 25-39	2 741	180	2 221	2 254	19	-1
Age 40-44	3 404	283	2 695	2 595	21	4
Age 45-64	18 518	1 438	13 471	12 004	27	11
Total	25 067	1 917	18 713	17 242	25	8

Number of simulated hospitalization days in the period of interest for different age groups in the four scenarios 1) without any vaccination in the period, 2) Full vaccine coverage in the whole period, 3) Actual vaccine roll-out, and 4) Alternative vaccine roll-out

## Discussion

Our study shows that the hospitalization days increased by 0.96 percentage points the first week and 1.57 percentage points the second week after date of confirmed COVID-19 for unvaccinated individuals. For individuals vaccinated with two doses of a COVID-19 vaccine, the corresponding increase was 0.46 the first week after the date of confirmed COVID-19 and 0.32 the second week. The difference between the youngest and oldest age groups was at its greatest in week two after positive test, where the number of hospitalization days was 50 (4/0.08) times higher for those aged 45-64 compared to those aged 18-24. There was no increase in hospitalization days after vaccination.

Conservative simulation results show that the actual vaccine roll-out reduced hospital days with 25 percent. Our results also show that an alternative vaccine roll-out that solely prioritizes by decreasing age could potentially have reduced hospitalization days with an additional 8 percent compared to the actual vaccine roll out. However, there are several other good reasons why age should not be the only priority criterion when distributing vaccines. Vaccination of health care workers was prioritized due to their crucial role in keeping the health care system operational, and medical risk groups were prioritized as they were more likely to get severe COVID-19 complications if infected. This simple simulation does not capture such priorities. Further research could also evaluate vaccine strategies that prioritize by other characteristics that are correlated with higher probability of being infected and/or higher probability of being hospitalized.

Our results both confirm and shed new light on the degree to which vaccination has relived the capacity strain on hospitals. A modelling study based on individuals in New York City used an age-stratified agent-based model of COVID-19 to simulate the counterfactual scenario of no vaccination and compared the resulting disease burden with the number of cases, hospitalizations and deaths reported under the actual pace of vaccination. Without vaccination, they projected that the total numbers of hospitalizations and deaths would have been over 2-fold higher than what was reported in the period of study [15].

To our knowledge this study is the first to explore to what degree vaccination of different age groups have relived the capacity strain on hospitals using nationwide observational data to estimate causal effects of vaccination on hospitalization days and incorporating these estimates into a simulation model. For the government and health authorities, our findings primarily show that ensuring vaccine uptake in the population aged above 45 is the key strategy in preventing future capacity constraints on hospitals. Important strengths of our study include the large sample size, the inclusion of both unvaccinated individuals with confirmed COVID-19, vaccinated individuals with confirmed COVID-19 individuals vaccinated with COVID-19 and vaccinated individuals with confirmed COVID-19, as well as the use of three comparison groups. Another strength is the use of routinely collected, daily nation-wide data from health registers that are mandated by law. Our methodological approach, where we studied acute overnight hospitalizations both before and after date of confirmed COVID-19 or vaccination also add to the strengths of the study, as it allowed us to provide estimates of what may happen to hospital capacity in the absence of vaccination, while controlling for time-variant confounders, such as seasonal changes to health care use that impact both groups to the same extent. We also incorporate these estimates into a simulation model that allow us to predict hospitalization days in different vaccine prioritization scenarios.

However, there are also certain limitations to our analysis. First, the vaccine should not be administered to individuals with infection and fever on or before the vaccination day, leading to selection of somewhat healthier individuals in the treatment group containing individuals vaccinated for COVID-19. We have therefore excluded observations 10 days prior to vaccine and 5 days after. Additionally, individuals who are eligible for the comparison groups are always infected/vaccinated later than those in the treatment group. However, it is not random who are infected/vaccinated first. If factors associated with time of infection/vaccination are also correlated with the probability of being hospitalized after infection, this could lead to bias in our estimates. For instance, individuals in medical risk groups were prioritized for vaccination, and they also had a higher underlying probability of hospitalization after COVID-19 infection. We addressed this when constructing the treatment group of vaccinated individuals by excluding the first 20 percent of the vaccinees. Second, this study does not consider waning immunity of the vaccine. Since we only study a limited time period this is probably of minor importance. However, countries including Norway have already started vaccination with a third dose, and waning immunity is therefore important when evaluating different vaccine strategies going forward. Additionally, in this study, we estimated the individual effect of vaccination on hospitalizations. However, the total effect of vaccination also contain positive externalities, such as reduced spread of infection and herd immunity that comes in addition to the individual effect. Vaccination can also help alleviate strict infection prevention and control measures kept in place to avoid strain on hospital capacity. More research is needed to understand the overall effects of vaccination. Our simulation model used data from before January 2021 to predict COVID-19 infection rates and hospitalizations from January 1^st^ to October 30^th^, 2021, which implicitly assumes that factors affecting the probability of COVID-19 infection and post-infection hospitalizations remain similar over time. This is, however, a strong assumption, as factors such as new virus variants, changes in infection control measures and vaccine effectiveness due to vaccine waning did vary over time. Finally, the simulation design is based on observed data and does not account for non-linear or dynamic effects, like e.g., poorer treatment due to operating closer to capacity limits, which might again lead to even more strain at the healthcare system, and so on.

### Concluding remarks

By studying health care use after COVID-19 and vaccination, we have shown that the hospitalization days was highly relived by vaccination, especially by vaccination of individuals aged 45-64. Our study thus illustrates that the individual utility of vaccination is highest among the elderly population and that vaccination of the elderly population is the key strategy in preventing hospital collapse. By incorporating causal estimates into a simulation model, we have also illustrated how simulation models can be used to evaluate alternative vaccine strategies. The simulation results shows that the observed vaccination in this age group averted more than 6 000 hospital days compared to the case of no vaccination. On the other hand, with full vaccine coverage in this age group, close to 19 000 hospital days could have been averted, although not realistic in this time period.

## Supplementary Information

**Additional file 1:** **Supplemental Figure S1.** Vaccine roll-out scenarios. **Supplemental Figure S2.** Impact of vaccination on hospitalization days. Supplemental Table S1. Impact of confirmed COVID-19 and vaccination on hospitalization, by age groups. **Supplemental Figure S3.** Impact of vaccination on hospitalization days by age. **Supplemental Figure S4.** Simulated infection rates. Simulated infection rates under the assumption that no one is vaccinated based on aggregate data from October 1^st^, 2020 to October 30^th^, 2021 for the age groups specified in the analysis. We control for age group, vaccine share within age groups and infection-rates in the preceding week, as well as calendar-week fixed effects.

## Data Availability

The data that support the findings of this study are available from Norwegian Directorate of Public health (Norwegian Patient Register), Norwegian Institute of Public Health (Norwegian Immunization Registerand Norwegian Surveillance System for Communicable Diseases) and Norwegian Tax Administration (National Population Register) but restriction apply to the availability of these data, which were used under license for the current study, and so are nor publicly available. Data are however available from the authors upon reasonable request and with permission of Norwegian Directorate of Public health Norwegian Institute of Public Health and Norwegian Tax Administration.

## Abbreviation

COVID-19: Corona virus disease 2019

## Footnotes

1 The establishment of an emergency preparedness register forms part of the legally mandated responsibilities of The Norwegian Institute of Public Health during epidemics. The project was approved by the Ethics Committee of South-East Norway confirmed (June 4th, 2020, #153204), which confirmed that external ethical board review was not required.
