# Supplementary material for: Has vaccination alleviated the strain on hospitals due to COVID-19? A combined difference-in-difference and simulation approach
Source: BMC Health Serv Res. 2022 Sep 21;22:1183. doi: 10.1186/s12913-022-08541-x (PMC9490737; doi:10.1186/s12913-022-08541-x)
Supplement: Supplementary file 1 — Additional file 1: Supplemental Figure S1. Vaccine roll-out scenarios. Supplemental Figure S2. Impact of vaccination on hospitalization days. Supplemental Table S1. Impact of confirmed COVID-19 and vaccination on hospitalization, by age groups. Supplemental Figure S3. Impact of vaccination on hospitalization days by age. Supplemental Figure S4. Simulated infection rates. Simulated infection rates under the assumption that no one is vaccinated based on aggregate data from October 1st, 2020 to October 30th, 2021 for the age groups specified in the analysis. We control for age group, vaccine share within age groups and infection-rates in the preceding week, as well as calendar-week fixed effects. [file 12913_2022_8541_MOESM1_ESM.docx]

**Details on propensity score matching**

To mimic a situation with quasi-experimental as-if random distribution COVID-19 and vaccines we constructed comparison groups using the propensity score matching method. We matched each treated individual to two untreated individuals (the inclusion criteria for being in the treatment group differs between the different treatment groups as explained in the Table 1). The propensity scores were created via a logit model that estimated the likelihood of testing positive for COVID-19 and receiving a vaccine, imitating a randomized distribution of COVID-19 and vaccines. We estimated the probability of A) testing positive for COVID-19 while unvaccinated, B) testing positive for COVID-19 after two doses of vaccine C) receiving vaccine, by using the following covariates: age (18-24, 25-39, 40-44, 45-64); sex; whether the individual is foreign born or not, and county of residence.

We used the nearest-neighbor (NN) method to balance the treatment and comparison groups (Stuart, 2010), which minimizes the absolute difference between the propensity scores of the vaccinated individual and its two controls. The maximum distance between potential neighbors were set to 0.001. To further increase the quality of our matches, we matched using replacement which implies that individuals in the target set could be matched to treated individuals more than one time. Duplicates in the comparison group means that the data is no longer independent. We therefore adjusted for the dependency in our data using clustered standard errors in the main analysis.

**Supplemental Figure S1: Vaccine roll-out scenarios**


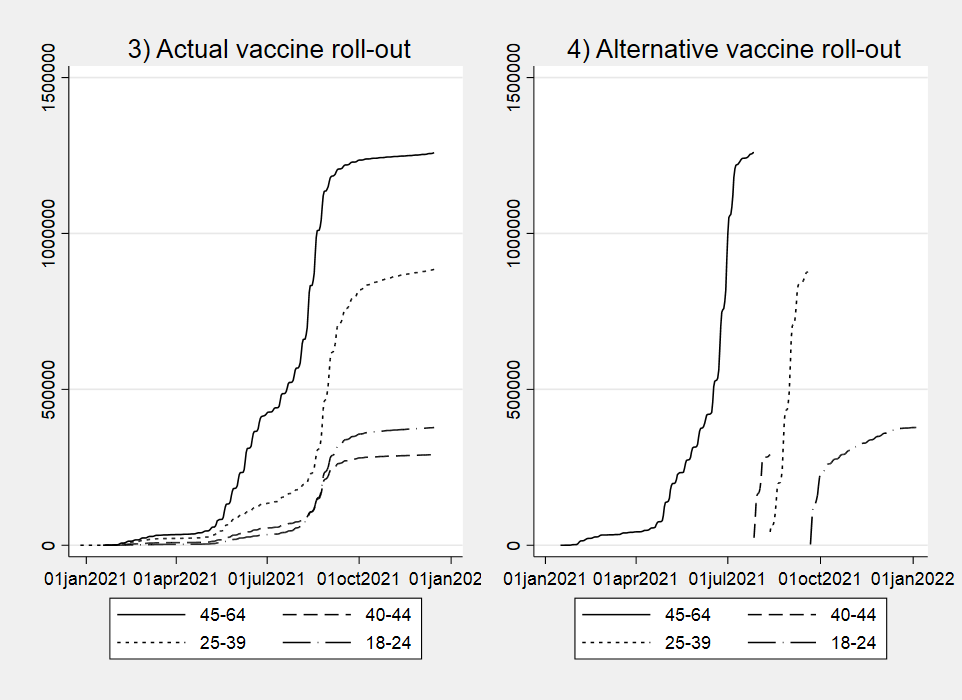
**Supplemental Figure S1 legend**: Cumulative number vaccinated with two doses in each age category for the scenario with actual vaccine roll-out (scenario 3) and scenario with an alternative vaccine roll-out (scenario 4) where all individuals in the age category 45-64 is prioritized before those aged 40-44 and so on.

**Supplemental Figure S2: Impact of vaccination on hospitalization days**
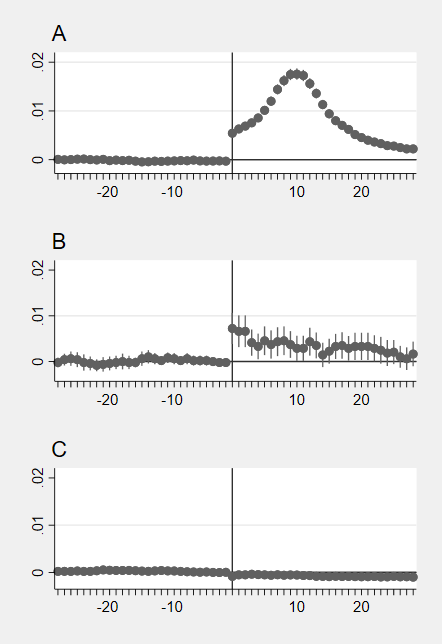


**Supplemental Figure S2 legend**: Estimated daily difference (95% CI) in acute overnight hospitalizations prior to and after treatment date for A) confirmed COVID-19 for the unvaccinated; B) confirmed COVID-19 for the vaccinated; C) First dose of vaccination. Relative day -1 is reference day for all regressions. Standard errors are clustered on individuals.

**Supplemental Table S1: Impact of confirmed COVID-19 and vaccination on hospitalization, by age groups**

|  | A) COVID-19 (unvaccinated)  b/(se) |  | B) COVID-19 (vaccinated)  b/ (se) |  | C) Vaccination  b/ (se) |
| --- | --- | --- | --- | --- | --- |
| **Age 18-24** |  |  |  |  |  |
| Week 1 | 0.14^***^ |  | 0.10 |  | -0.04 |
|  | 0.030 |  | 0.138 |  | 0.037 |
| Week 2 | 0.08^**^ |  | 0.02 |  | -0.05 |
|  | 0.031 |  | 0.155 |  | 0.042 |
| Week 3 | 0.04 |  | -0.10 |  | -0.03 |
|  | 0.026 |  | 0.150 |  | 0.048 |
| Week 4 | 0.02 |  | 0.02 |  | -0.10 |
|  | 0.025 |  | 0.128 |  | 0.063 |
| N: | 2 245 112 |  | 71 684 |  | 1 089 902 |
| **Age 25-39** |  |  |  |  |  |
| Week 1 | 0.69^***^ |  | -0.06 |  | -0.11^***^ |
|  | 0.048 |  | 0.146 |  | 0.025 |
| Week 2 | 0.75^***^ |  | 0.08 |  | -0.15^***^ |
|  | 0.056 |  | 0.175 |  | 0.029 |
| Week 3 | 0.22^***^ |  | 0.18 |  | -0.19^***^ |
|  | 0.035 |  | 0.108 |  | 0.033 |
| Week 4 | 0.09^**^ |  | 0.20 |  | -0.22^***^ |
|  | 0.030 |  | 0.197 |  | 0.036 |
| N: | 3 162 773 |  | 99 970 |  | 3 408 245 |
| **Age 40-44** |  |  |  |  |  |
| Week 1 | 0.95^***^ |  | 0.73^*^ |  | -0.07 |
|  | 0.108 |  | 0.353 |  | 0.036 |
| Week 2 | 1.47^***^ |  | 0.08 |  | -0.11^**^ |
|  | 0.146 |  | 0.141 |  | 0.041 |
| Week 3 | 0.37^***^ |  | -0.00 |  | -0.14^**^ |
|  | 0.085 |  | 0.000 |  | 0.051 |
| Week 4 | 0.20^**^ |  | -0.00 |  | -0.08 |
|  | 0.077 |  | 0.000 |  | 0.049 |
| N: | 885 691 |  | 46 739 |  | 1 016 265 |
| **Age 45-64** |  |  |  |  |  |
| Week 1 | 2.04^***^ |  | 0.80^**^ |  | -0.06^*^ |
|  | 0.093 |  | 0.244 |  | 0.023 |
| Week 2 | 4.00^***^ |  | 0.62^**^ |  | -0.06^*^ |
|  | 0.141 |  | 0.231 |  | 0.024 |
| Week 3 | 1.85^***^ |  | 0.59^*^ |  | -0.08^**^ |
|  | 0.101 |  | 0.261 |  | 0.026 |
| Week 4 | 0.84^***^ |  | 0.25 |  | -0.07^**^ |
|  | 0.076 |  | 0.226 |  | 0.026 |
| N: | 2 428 974 |  | 194 361 |  | 4 821 098 |

*Notes: Difference-in-difference estimated for the change in acute overnight hospitalizations before and after treatment date. Standard errors (se) are clustered on individuals. The pre-period is reference period in all regressions. Significance levels: *<0.1, **<0.05, ***<0.01*

**Supplemental Figure S3: Impact of vaccination on hospitalization days by age**


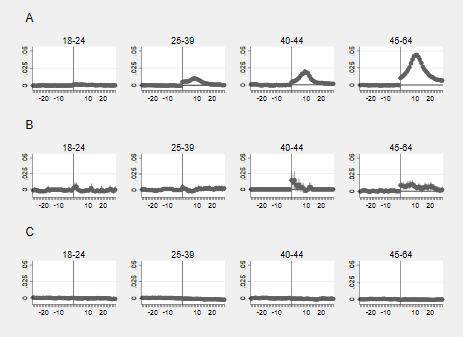


**Supplemental Figure S3 legend**: Estimated daily difference (95% CI) in acute overnight hospitalizations by age prior to and after treatment date for A) confirmed COVID-19(unvaccinated) B) testing positive for COVID-19 (vaccinated with two doses) and C) vaccinated first dose. Relative day -1 is reference day for all regressions. Standard error is clustered on individuals.

**Supplemental Figure S4: Simulated infection rates**


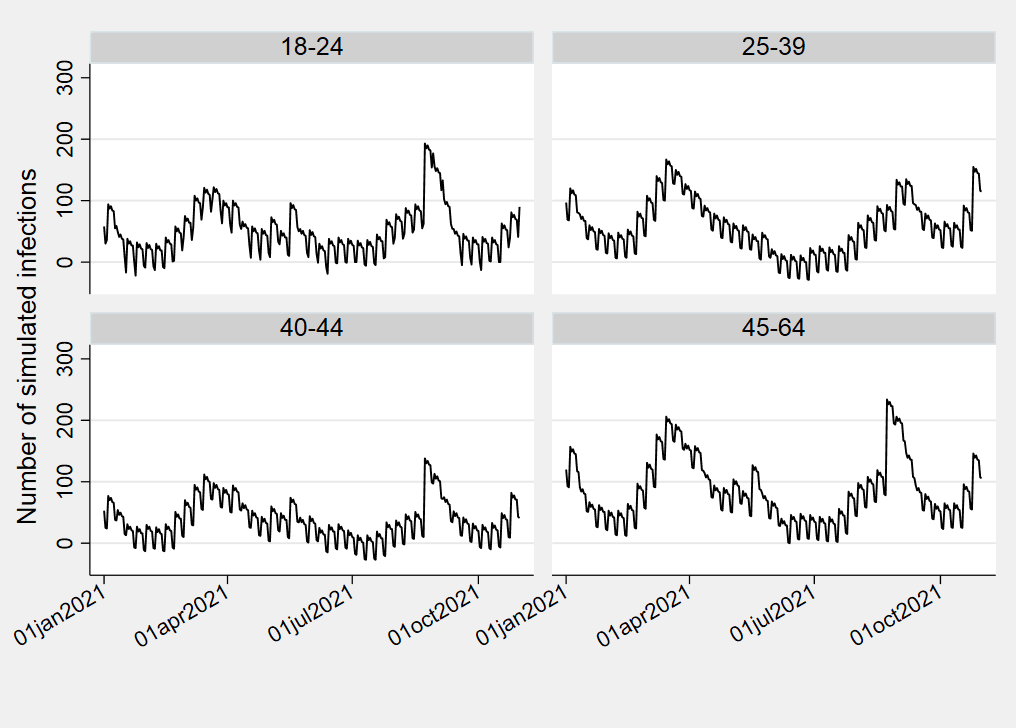


**Supplemental Figure S4 legend:** Simulated infection rates under the assumption that no one is vaccinated based on aggregate data from October 1^st^, 2020 to October 30^th^, 2021 for the age groups specified in the analysis. We control for age group, vaccine share within age groups and infection-rates in the preceding week, as well as calendar-week fixed effects.

**References**

Stuart, E. A. (2010). Matching methods for causal inference: A review and a look forward. *Statistical science: a review journal of the Institute of Mathematical Statistics*, *25*(1), 1.
